# Evaluation of the Intussusception Risk after Pentavalent Rotavirus Vaccination in Finnish Infants

**DOI:** 10.1371/journal.pone.0144812

**Published:** 2016-03-07

**Authors:** Tuija Leino, Jukka Ollgren, Nina Strömberg, Ulpu Elonsalo

**Affiliations:** 1 Department of Health Protection, National Institute for Health and Welfare (THL), Helsinki, Finland; 2 Department of Infectious Diseases, National Institute for Health and Welfare (THL), Helsinki, Finland; The Australian National University, AUSTRALIA

## Abstract

**Background:**

An association between rotavirus immunisation and intussusception (IS) has been suggested with present rotavirus vaccines in post-licensure studies. In Finland, rotavirus vaccination programme was implemented in September 2009 using a 2, 3, and 5 months schedule with the pentavalent rotavirus vaccine. By the end of 2013, it is estimated that 719 000 rotavirus vaccine doses have been given in the national programme of which 240 000 were first doses. Nationwide register allows us to evaluate the association between rotavirus vaccination and IS.

**Methods and Materials:**

Cases of IS diagnosed during 1999–2013 were identified from National Hospital Discharge Register. All cases under 250 days of age diagnosed during 2009–2013 were confirmed by reviewing medical charts. Self-controlled case-series method was used to assess the risk of IS during 1–21 days compared to 22–42 days post vaccination.

**Findings:**

In register data the relative incidence of IS at 2 months of age between the post and pre vaccination era was 9.1 (95%CI 2.0–84.3). We identified 22 verified cases with date of admission less than 43 days after any of the three rotavirus vaccine doses. The incidence of IS in the risk period after the 1st dose relative to the control period was 2.0 (95% CI 0.5–8.4; p = 0.34.) Number of excess IS cases per 100 000 first vaccine doses was therefore estimated to be 1.04 (95% CI 0.0–2.5), i.e. one additional IS case per 96 000 first doses of rotavirus vaccine (95% CI 54 600 to ∞). There was no risk detected after 2nd and 3rd doses.

**Conclusion:**

The finding is in line with the recent published estimates. The benefits of rotavirus immunisation programme outweigh possible small risks of intussusception.

## Introduction

Already for more than a decade, the link between rotavirus vaccines and intussusception (IS), a bowel syndrome where a segment of the small intestine invaginates within a more distal bowel segment, has been under particular scientific interest. After an association was found between the tetravalent rhesus reassortant rotavirus vaccine (RotaShield®) and intussusception in 1999, the manufacturer withdrew the vaccine from the market [1].

Due to this, the two vaccines currently widely used in national programmes underwent extensive safety studies before licensure [2, 3]. Powered to detect significant differences in the rare safety event IS, the pre-licensure clinical trials for both vaccines involved more than 60 000 infants each. In such studies, a risk of the magnitude seen after the first generation rotavirus vaccine 1/4000 to 1/10 000, should have been observed [4]. However, no association between IS and rotavirus vaccines tested was detected, as cases were distributed evenly in rotavirus vaccinated and placebo recipient children[2, 3]

Thereafter several reviews of IS incidence in populations either with or without rotavirus immunisations have been published [5–7]. These have come to a conclusion, that the incidence of IS differs greatly between countries, and it is to be surveyed carefully after rotavirus vaccine introduction [6, 8]. Furthermore, in some recent post-licensure studies, an increased risk of IS has been observed regardless of the rotavirus vaccine used [9–15], although some contradictory findings have also been reported [16, 17]. The estimates presented in these studies for the number of vaccinations needed to produce 1 additional IS case have ranged from 14 000 to 199 000 doses.

In Finland, with a birth cohort of 59 000, rotavirus vaccine was added to the national immunisation programme already in September 2009. More than 700 000 doses rotavirus vaccine given as well as national Hospital Discharge Register capturing IS cases, allow us to evaluate the association between rotavirus vaccination and IS in Finnish infants.

## Materials and Methods

### Study population and design

The infants born on 1^st^ of July 2009 or after have been offered the pentavalent rotavirus vaccine (RotaTeq^R^) at 2, 3 and 5 months of age. All IS cases of children under 1 year of age treated in the Finnish public hospitals from 1999 until the end of year 2013 were utilised in the analyses.

As a preliminary ecological analysis, based on plain register data, we compared the incidence of IS in infants less than 1 year of age prior rotavirus vaccine era to the incidence during the national rotavirus immunisation programme. As the national immunisation register is only presently being formed, vaccinated and unvaccinated cohorts during the immunisation programme could not be reliably defined retrospectively. Therefore, for the final analysis we gathered all IS cases under 1 year of age, verified their immunisation records from the child welfare clinics directly and utilized self–controlled case-series (SCCS) design for examining the association between rotavirus vaccine and IS.

### Case finding and verification

Cases were primarily sought from the whole country using national Hospital Discharge Register (HILMO) data for years 1999–2013. This register contains all hospitalisations and secondary care outpatient visits such as emergency room visits and visits to paediatricians at a public sector. In the international classification of diseases the code K56.1 is used for IS in the tenth edition (ICD 10) which has been used in Finland since 1998. The code was searched from the primary diagnoses and duplicate visits/admissions during a 7 day period were united to form a case by using the social identity code unique for each child. A case was defined to have occurred at the day of the first admission.

Full-text medical records were collected from hospitals for all register based IS cases less than 250 days of age from 1^st^ of September 2009 until 31^st^of December 2013. This age limit was set as rotavirus vaccines should not have been given after 6 months of age. Cases were verified by two reviewers using the Brighton Collaboration case definition [18]. The latter reviewer was blinded to the vaccination status of each case. Cases were excluded if they did not fulfil the Brighton Collaboration case definition or if an alternative diagnosis had been made, for example based on surgery findings. All cases used in the primary analysis were confirmed to have the highest level of diagnostic certainty i.e. level 1 in the Brighton Collaboration classification [18].

Immunisation data for the individual IS cases were sought primarily from the register being presently constructed. If in the light of the immunisation register IS cases were unvaccinated, data was also sought directly from the child welfare clinic of each particular child.

### Ethics statement

This study was conducted on behalf of National Institute for Health and Welfare (THL) as a public health investigation and it is approved by the THL Ethics Committee (Dnr THL/1664/ 6.02.00/2011). For the individual based data a separate approval was applied and issued (DNR THL/95/6.02.00/2014). Furthermore, the patient records were anonymized prior to the analysis.

### Statistical methods

We used the self-controlled case-series (SCCS) method [19] to estimate the relative incidence of intussusception during the pre-defined risk periods of 1 to 7 and 1 to 21 days after vaccination (vaccination at day 0) and control interval 22 to 42 days post-vaccination. These risk periods were chosen based on the assumption of timing of the possible IS cases after vaccination. The same periods have also been utilised in other recent studies [11, 14] which allow direct comparison. Only IS cases which occurred within 42 days after rotavirus vaccination were included. As a sensitivity analysis, a risk window 1–15 post-vaccination days was also utilised.

Self-controlling restricts both known and unknown person-related confounding, such as sex and predisposing factors, effectively [20]. Furthermore, self-controlled analyses minimise the effort needed in exposure verification as only persons with outcome of interest are to be considered instead of wide unexposed and exposed cohorts. As the risk of intussusception among infants is, however, very age-dependent, we could not directly compare the incidences during the pre-defined intervals. To adjust for the known increase in the risk of intussusception during the ages from 2 to 6 months we used Hospital Discharge Register based IS incidence data from years 1999–2005 in Finland. During those years rotavirus vaccines were not available. Technically, we used conditional Poisson regression model with an offset term to adjust for the differential risk of intussusception according to ages in the risk and control intervals. The age-dependency was described by a quadratic function.

We also present the average attributable risk on the basis of the observed age distribution of the vaccinated children. The attributable risk was calculated as the number of excess cases of intussusception per 100 000 vaccine doses administered, according to the formula:

$\frac{100,000\times N_{w}\times \left(1-\frac{1}{rr}\right)}{D_{v}}$, where N_w_ is a number of cases in the risk window, rr is the relative risk (risk period/control period) and D_v_ is a number of vaccine doses given.

For the number of vaccine doses administered we used official vaccination coverage estimates. The vaccination coverages in Finland have been surveyed by taking a random sample of 1 000 children at 3 years of age and verifying their immunisation records directly from the health baby clinic of each particular child. Based on the last coverage survey carried out 2013–4 among infants born 2009, rotavirus immunisation coverage has been 93.2% for all three doses, and 94.0% for both the 1st and 2nd doses. (http://www.thl.fi/fi/web/rokottaminen/kansallinen-rokotusohjelma/rokotuskattavuus/pikkulasten-rokotuskattavuusverkkosivut)

## Results

### Register study

**Fig 1:**
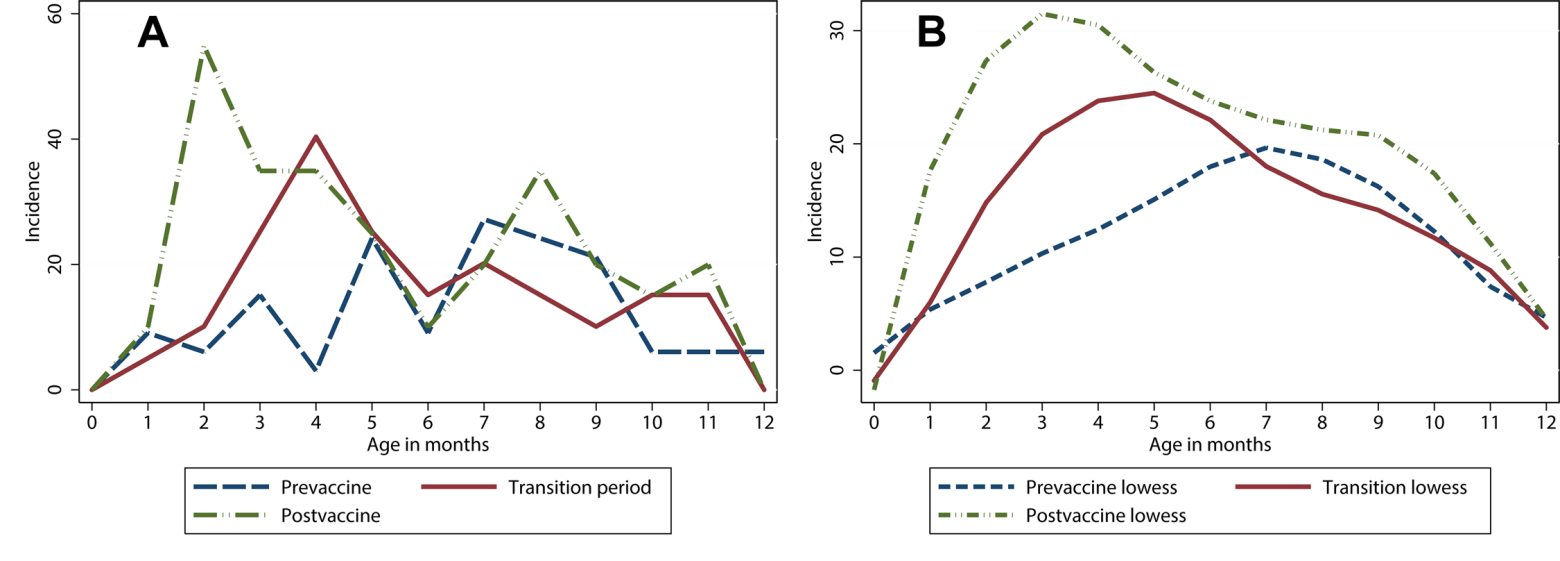
The incidence of intussusception at three different periods. a) The crude and b) the smoothed incidence of intussusception per 100 000 person years during pre-vaccination years (1999–2005) with blue line, when vaccine was sold (2006–2009) with red line, and after the immunisation programme implementation (2010–2013) with green line by month of age for children less than 1 year in Finland. Fig is based on National Hospital Discharge Register data, and case verification has not been performed. Cases were referred to as 1 month of age from 15 days to 45 days of age, 2 months from 46 to 75 days of age etc.

In the Hospital Discharge Register during years 1999–2013, a total of 149 IS cases under 1 year of age were identified. Of these, 60 were observed during the 4 years and 4 months (September 2009- December 2013) of the national infant rotavirus programme, 52 IS cases were reported during the 7 years prior to the vaccines were available (January 1999-December 2005), and 37 cases during the 3 years and 8 months while the vaccine was available in the market but not in the national programme (2006- August 2009). The corresponding cumulative incidences at 1 year of age during the three periods were 7.4 cases per year prior, 10.1 cases per year while vaccine was sold and 13.8 annual cases during the universal vaccination programme. The register based IS incidences, especially the age-specific shape of incidence curves, were different between the three periods (Fig 1A and 1B). The IS incidence based on Hospital Discharge Register data was higher during the vaccination programme, compared to the era prior to vaccinations, especially at 2 months of age. The relative incidence rate ratio at 2 months of age between the post and pre vaccine era as seen in the register data was in fact 9.1 (95%CI 2.0–84.3).

All IS cases under 1 year of age during the pre-vaccine years 1999–2005 were utilized to illustrate the age-specificity of IS in Finland. Based on these 52 cases, the mean pre-vaccine incidence was 12.1/ 100 000 person years (95%CI 9.2–15.9) and the maximum incidence was 27.2 (95% CI 14.2to 52.3) per 100 000 at 7 months of age.

### SCCS with verified IS cases only

**Fig 2:**
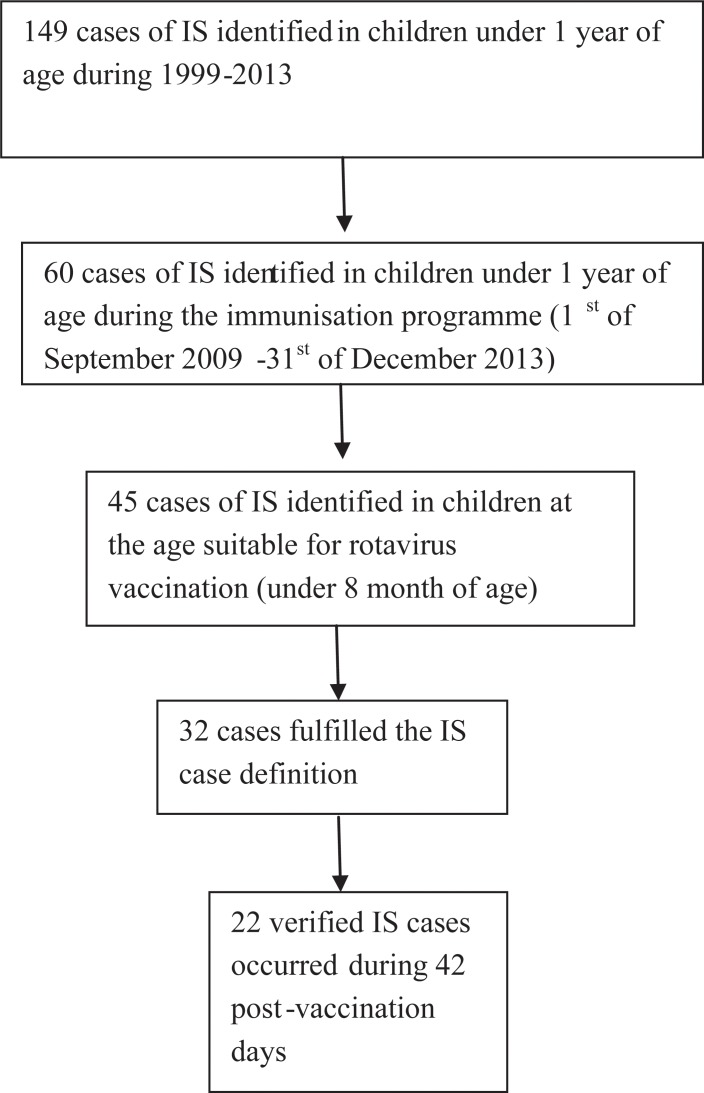
Selection of data for self- controlled case-series analysis. Cases of IS were primarily identified in the Hospital Discharge Register. Case verification was performed for cases eligible for rotavirus vaccine (under 8 months of age) which occurred during the immunisation programme. Case verification was carried out using Brighton collaboration case definition [18].

Case verification was performed to all 45 IS cases under 250 (~ 8mo) of age during the national rotavirus immunisation programme 1^st^ September 2009 – 31^st^ December 2013 (Fig 2). During the verification 13 (29% of cases) were discarded. The alternative diagnoses were: 3 dolores abdominis, 3 blood in feces, 2 constipations, 1 inguinal hernia, 1 vomiting, 1colitis, and 2 serious congenital gastrointestinal malformations. From the 32 verified IS cases four children were unvaccinated and six had IS either before the first vaccine dose or more than 42 days after any of the vaccine doses.

**Fig 3:**
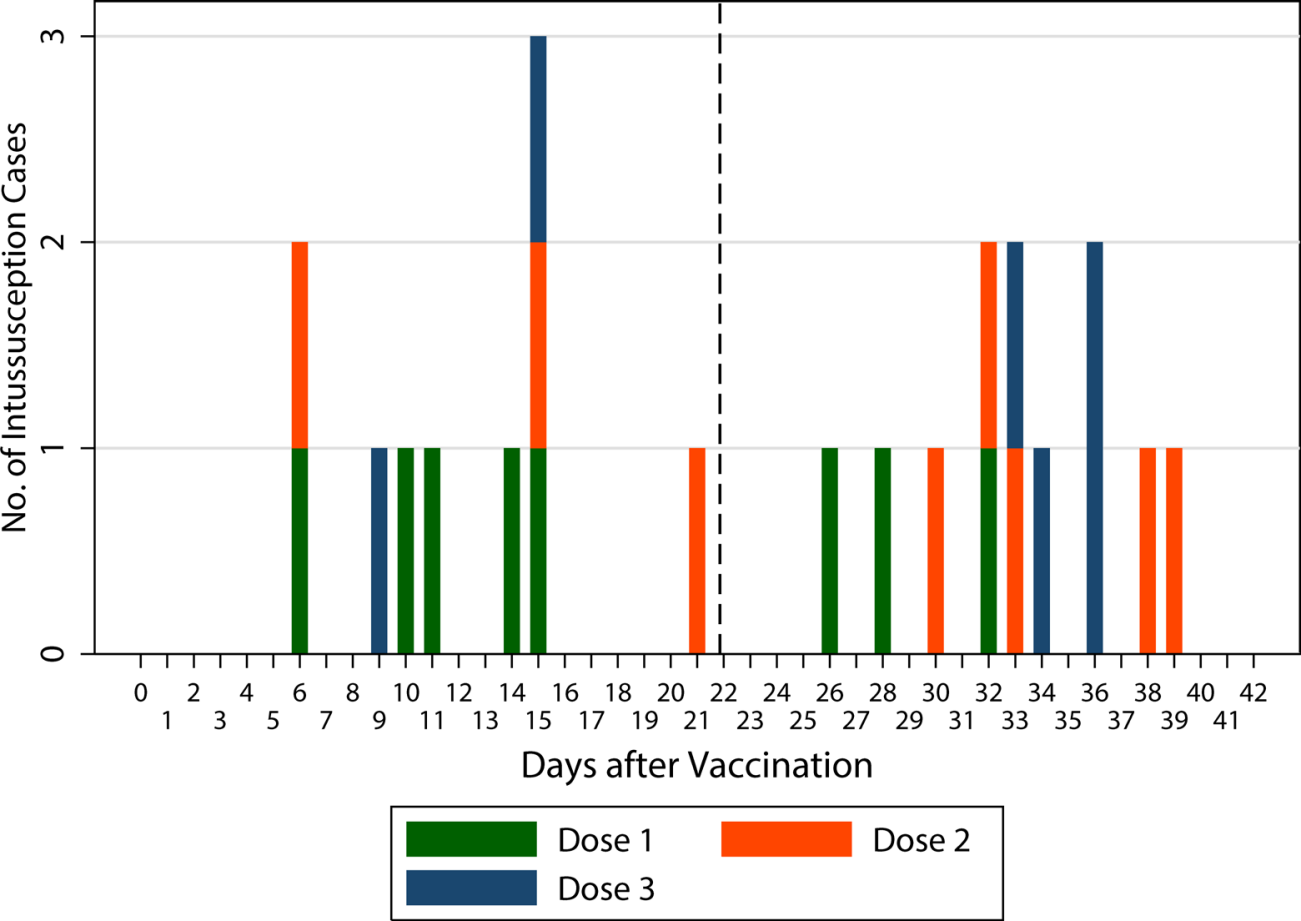
Distribution of intussusception cases according to day of admission. Cases which have occurred after the first vaccine dose are marked green, after the second dose red and after the third dose blue. Vertical broken line separates the pre-specified risk and control intervals.

In the SCCS analysis, we had 22 vaccinated cases with date of admission less than 43 days after any of the three vaccine doses (Fig 3). There was only one case during the 1 to 7 days risk period after the first dose and one after the second dose. Therefore, only the risk period of 1–21 days was used in the main analysis. There were 5 cases during this period. The point estimate of the incidence of IS in the risk period after the first dose relative to the control period was 2.0 (95% CI 0.5–8.4; p = 0.34). Based on vaccination coverage survey, it was estimated that there were all together 719 000 rotavirus vaccine doses given during the national programme of which 240 000 were first doses.

Number of excess IS cases per 100 000 first doses was therefore estimated to be 1.04 (95% CI 0.0–2.5), in other words, one additional IS case per 96 000 first doses of rotavirus vaccine (95% CI 54 600 to ∞). In Finland, that would mean less than 2 excess cases per 3 years of immunisation programme.

However, as there was 1 case whose follow up was censored due to a second vaccine dose (38^th^ day post vaccination) we also calculated the risks using another risk period, 1–15 days post vaccination. We compared the incidence during this period to the incidence during 16–30 post-vaccination days. There were 5 IS cases during this risk period, and the point estimate of the incidence rate ratio was thus 2.8 (95% CI 0.5–14.5). With this estimate, rotavirus vaccination could be expected to lead to 1.3 additional cases of IS per 100 000 first doses given i.e. 1 additional case per 74 600 (95% CI 51 600 to ∞) first doses of pentavalent rotavirus vaccination.

There was no risk detected after the second and third doses: the relative numbers of cases (as is seen in the Fig 3) are 3 divided by 5 and 2 divided by 4, respectively. The age-correction during the 42 days, being less than one and half is not strong enough to make such “protective”incidence rate ratios larger than one.

## Discussion

In this post marketing study an estimate for the number of additional IS cases due to the rotavirus vaccination programme using pentavalent vaccine was estimated by self-controlled case-series analysis. Cases were identified from the national Hospital Discharge Register, case verification was carried out using patient charts and immunisation data was confirmed from health baby clinics directly. According to this study, there was one excess case of IS per 96 000 first rotavirus vaccine doses given. As our estimate is based on a small number of cases, confidence intervals are wide. However, as the original risk of IS is very low, even by selecting the risk estimate from the high end of our confidence interval, produces still practically only 1 additional case per 54 600 first doses given.

Our risk estimates are in line with the other recently published findings on pentavalent vaccine. A study utilising Vaccine Safety Datalink material from United States reported one additional case per 199 000 first doses given (95% CI 1/56 000- ∞) [13]. In another American study covering half a million first doses gave a risk estimates of 1/65 000 and 1/80 000 for self-controlled risk interval and cohorts analyses, respectively (95% CI for SCCS 1/31 000–1/519 000) [14]. When data were gathered via Vaccine Adverse Event Reporting System (VAERS) and analysis used also self-controlled risk period setting, the additional risk was 1/135 000 (95% CI 1/58 000–1/417 000) [12]. The biggest additional risk with pentavalent rotavirus vaccine, one IS case per 14 000 first vaccine doses, has been reported in an Australian study using also self–controlled analysis (95% CI 1/7 000–1/32 000) [11].

Our study suggests that there is a slightly increased risk of getting IS during the 21 days since the first vaccine dose. However, during the first post-vaccination week we did not find any particular risk of IS. This is somewhat unexpected as the first week after vaccination was the period of most pronounced risk both after RotaShield® [1] as well as after present rotavirus vaccines[9, 11, 14]. It is also the time when majority of adverse events are reported for example to VAERS system [12]. Furthermore, it corresponds to the period of peak intestinal replication of vaccine virus [21] [9, 12]. One explanation for the finding could be that children in Finland would seek care later during the course of IS compared to children in other countries. In the light of our material, this should not be the case: admission date is never later than the 3^rd^ day of symptoms. Treatment results among all IS cases in our material were also good, resection of any bowel segments were rarely needed (2/32 cases) with no deaths reported. Laparotomy was performed in 23 out of the 32 verified cases.

Further, the cases were listed by their day of admission, not by the day then the verified diagnosis was set, as was recommended by Brighton collaboration case definition [18]. The day of definite diagnosis is either the same or later date as admission, and using that could only have made the estimate more conservative (see Fig 3) if anything. We are therefore fairly confident that the IS cases reported to occur at the second week were not present during the first post-vaccination week.

The risk estimate based on SCCS analysis is considerably lower than the plain register data would have implemented. In register studies with historical comparison, it is possible that issues other than vaccination can have changed with time. Parents’ knowledge of IS, as a disease, could be one of such things. This is, in fact, very likely as when the rotavirus vaccination programme was initiated in Finland, most parents received a booklet mentioning IS and the increased risk of it linked with the earlier rotavirus vaccine. It is therefore possible that more infants with suspected IS were brought to emergency departments after vaccination programme introduction. Case verification is therefore essential. Uniform case definition [18] also enables international comparisons to be made between studies.

The main limitation of our study is the low number of IS cases. Finland is one of the countries with very low IS incidence, the cumulative incidence of intussusception among children has earlier been estimated to be 20 cases per 100 000 live births [22]. In our material the IS incidence among children less than one year of age is 13 per 100 000 while the corresponding international mean incidence has been estimated to be 74 per 100 000 ranging from 9 to 328 [5]. The background incidence during years 1999–2005 was very low: there were only 51 cases of IS under one year of age during the seven years. There are several known reasons for the low IS incidence. Finnish population is homogenous, and races with higher risk are rare [23]. Due to high standards of hygiene, enteritis due to pathogenic bacteria or helminthes, which are known to increase IS incidence [24], are rare, and moreover, cystic fibrosis is practically non-existing in Finland. Oral poliovirus vaccine has not been used during recent decades and maternal leave for nine months allow extended breastfeeding, which has been shown to reduce the risk of IS when compared to conventional baby formulas [23]. The observed lack of cases could also be due to a leaking register. However, the accuracy of the diagnoses as well as the registration coverage have been proven to be good in the Hospital Discharge Register [25] as more than 95% of discharges could be identified from the register. All in all, the low background incidence might limit the generalisability of our results to countries with higher incidence.

Another issue to consider is the fact that we verified the diagnoses only for IS cases during the national rotavirus vaccination programme not prior that. The background rate of IS based on these un-cleaned historical data could be artificially too high. The post-exposure risk estimates based on non-validated historical cohorts are thus prone to be underestimated. However, in SCCS the historical data were used only for illustrating the shape and extend of age-dependency of IS incidence. If the curve rises too steep due to non-validated data, we would in fact expect more cases in our control period, where children are older. As we use the relative incidence between risk and control periods, our risk estimate due to a too steep age dependency curve, in fact, overestimates the risk. In any case, the incidence prior immunisation programme has been very low, and therefore large effects, to any direction, are unlikely.

We had selected the periods of interest beforehand based of literature [11, 14] to allow direct comparison. As our vaccination programme is at 2, 3, and 5 months of age, a shorter period would have been more appropriate. We therefore analysed the result utilising a risk period of 1–15 days in relation to a control period 16–30 days post-vaccination. That did not change the result considerably.

The major strength of this study is that the ascertainment of events is independent of vaccination history. In other words, events were as probable to be ascertained anytime which is not the case in data collected using adverse event databases. Independent ascertainment is a prerequisite for traditional SCCS analysis which is, in fact, the preferable analysis for our data. It is economical and it controls for time-fixed confounding efficiently. Furthermore, when the risk period is short and vaccination coverage very high the decline in the relative efficiency for the SCCS to the cohort method is small [26]. When estimating relative incidences little info is missed when non-cases are ignored [20, 27].

The actual mechanism how rotavirus vaccine would lead to IS is unclear. It has been hypothesized that at least the earlier rotavirus vaccine RotaShield® caused reactive lymphoid hyperplasia, which acted as a leading point in IS formation [21]. It has also been questioned whether the increased risk of IS after vaccine doses is, in fact, true increase of IS or merely an earlier occurrence (triggering) of IS in infants among whom it would have occurred later in infancy in the absence of rotavirus vaccination [1, 9, 11, 28, 29]. Due to our very low incidence both prior and post immunisation programme introduction, our findings cannot rule out either of the presented theories. However, if rotavirus vaccines would cause IS as such, low background incidence should not have a major effect on the post-vaccination risks detected. This would indicate that some predisposing factors have a role in occurrence of IS linked with rotavirus vaccine. In any case, it remains to be seen if the rotavirus vaccine increases the number of IS cases or whether it only triggers cases.

## Conclusion

Based on more than 700 000 vaccine doses given during the national rotavirus immunisation programme we estimated that there was a very limited increased risk of IS associated with pentavalent rotavirus vaccine when the first dose was given. However, since the start of the national rotavirus vaccination program rotavirus disease and associated hospital treatments have been reduced by 80–90% [30–32]. Thus, the benefits of rotavirus immunisation programme clearly outweigh possible small risks of intussusception.
